# Differentiating tics from functional (psychogenic) movements with electrophysiological tools

**DOI:** 10.1016/j.cnp.2019.04.005

**Published:** 2019-06-28

**Authors:** Felipe Vial, Sanaz Attaripour, Mark Hallett

**Affiliations:** a National Institutes of Health, NINDS, Human Motor Control Section, Building 10, 9000 Rockville Pike, Bethesda, MD 20892, USA; b Universidad del Desarrollo, Facultad de Medicina Clínica Alemana, 5951, Av Vitacura, Vitacura, Región Metropolitana, Chile

**Keywords:** Functional movement, Psychogenic movement, Tic, Bereitschaftspotential

## Abstract

**Objective:**

We report on two patients that presented to our clinic with the differential diagnosis of functional movement disorder vs tic disorder.

**Case reports:**

The first patient is a 23-year-old woman with a 3-year history of repetitive involuntary neck flexion movements. The second patient is a 59-year-old man with a 10-month history of involuntary trunk jerks. In both cases, the polyelectromyographic study and the observation of Bereitschaftspotential helped us in making the final diagnosis.

**Significance:**

The use of electrophysiological tools can be helpful in making the distinction between functional movement disorders and tic disorders.

## Highlights

• Clinical observations alone often fail to discriminate functional jerky movements and jerks due to a tic disorder.
• Neurophysiological studies with EEG and polymyography assist with differentiating these conditions with more certainty.
• Further studies are needed for understanding the value, sensitivity and specificity of these techniques.

## 1 Introduction

Phenomenologically, functional movement disorders (FMD) are characterized as movements that are significantly altered by distraction and are clinically incongruent with movement disorders known to be caused by neurological diseases (Edwards et al., 2013). Tics are defined as sudden, rapid, recurrent, nonrhythmic motor movements or sounds. They are often preceded by a premonitory sensation that could be either a localizable sensation of discomfort in the region of the tic or a more generalized urge to move (Jankovic and Kurlan, 2011). They are considered to be voluntary movements made automatically so that volition is not ordinarily appreciated (Hallett, 2015).

Differentiating motor tics and FMD based on the clinical history and examination can be challenging because they can be similar in terms of the sudden onset, variability of movement distributions, distractibility, suggestibility, temporary remissions and waxing and waning courses (Edwards et al., 2013).

Here we report two patients who were referred to our clinic originally with the diagnosis of functional movement disorder, but the electrophysiological studies helped change the diagnosis to tic disorder.

## 2 Cases

### 2.1 Case 1

23-year-old woman presented with 3-year history of abnormal movements of the head and neck. Her neck movements were jerky, periodic and semi-rhythmic. The movements looked myoclonic. The head was pulled down symmetrically, but she reported episodes of sustained torsion of the head toward the right or left. Her jaw would briefly open during these movements.

Initially, the movements were less frequent but gradually became more prevalent. They were happening every 5 s when she was seen at our clinic. The movements caused extreme pain and discomfort and had affected patient's quality of life tremendously. The movements continued during early stages of sleep based on a video recorded by her family. On the first visit, she denied any preceding urge or any relief after the movements. She could suppress the movements by stiffening her neck muscles. The movements would subside while she was talking, singing or chewing. Physical or emotional stress made her movements worse. Alcohol worsened her movements to the point that she stopped drinking alcohol completely.

Work up including blood work for complete blood count, complete metabolic panel, Serum TSH, ceruloplasmin and serum copper had been all normal. MRI brain with and without contrast and MRI cervical spine were unremarkable. EMG of upper extremities was remarkable only for occurrence of myoclonic jerks. EEG showed no spikes or any other abnormalities previous to the movements.

She had been diagnosed with different conditions; e.g., focal or segmental myoclonus, cervical dystonia and also functional movement disorders. A trial of clonazepam did not provide any relief. Cyclobenzaprine was not tolerated by the patient due to drowsiness. She tried botulinum toxin injection three times with minimal benefit only and at times she experienced adverse effects like swallowing difficulty and neck extension weakness with head drop. As the clinical picture did not clearly favor any diagnosis, we performed a physiological study to characterize the movements and record the cortical event related potentials.

Surface EMG was recorded from anterior cervical muscle group, sternocleidomastoid (SCM), trapezius, levator scapulae and abductor pollicis brevis. EEG was record from C3, C4 and Cz. Bereitschaftspotential potentials (BP) were sought by back averaging the EEG to the beginning of her involuntary movement (50 trials) and also during a known voluntary movement (abduction of her thumb).

**Fig. 1:**
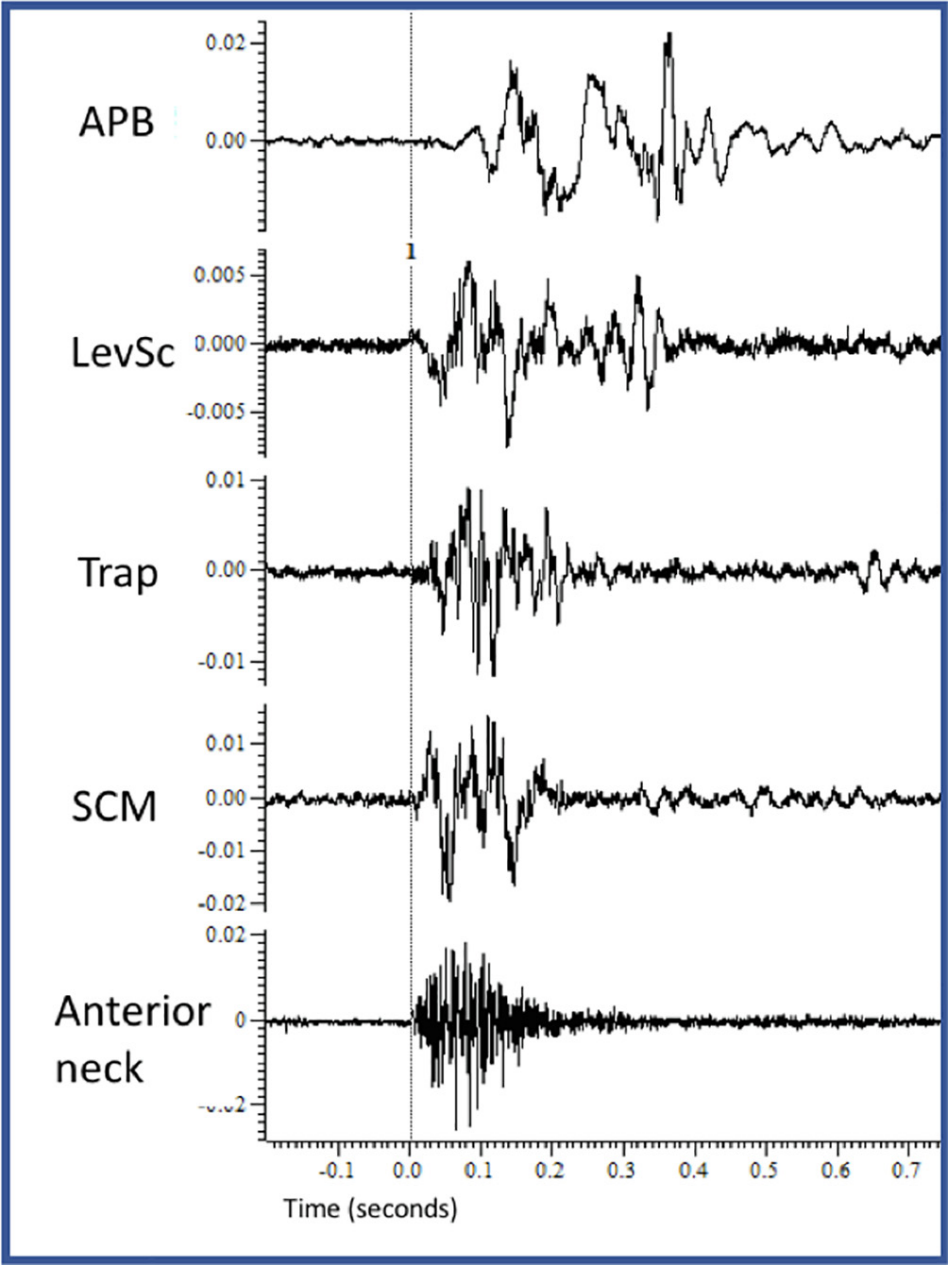
Case 1 EMG study. Back average of 50 trials. APB: Abductor pollicis brevis, LevSc: Levator scapulae; Trap: Trapezius, SCM: Sternocleidomastoid.

During the surface EMG study there was a consistent pattern of activation starting with almost simultaneous contraction of SCM and the anterior cervical muscles group in 200 ms bursts, followed by the trapezius and levator scapulae 20 ms later and then APB 65 ms later. The same pattern was repeated non-rhythmically with a frequency of approximately 12 per minute (Fig. 1).

When the patient was asked to try to sleep and the environmental noise and light were minimized, the spontaneous activity was reduced in frequency. The frequency of the movements increased when patient was asked to make voluntary movements at the same frequency as a metronome clicking at 1 and 2 Hz, but no entrainment was detected.

**Fig. 2:**
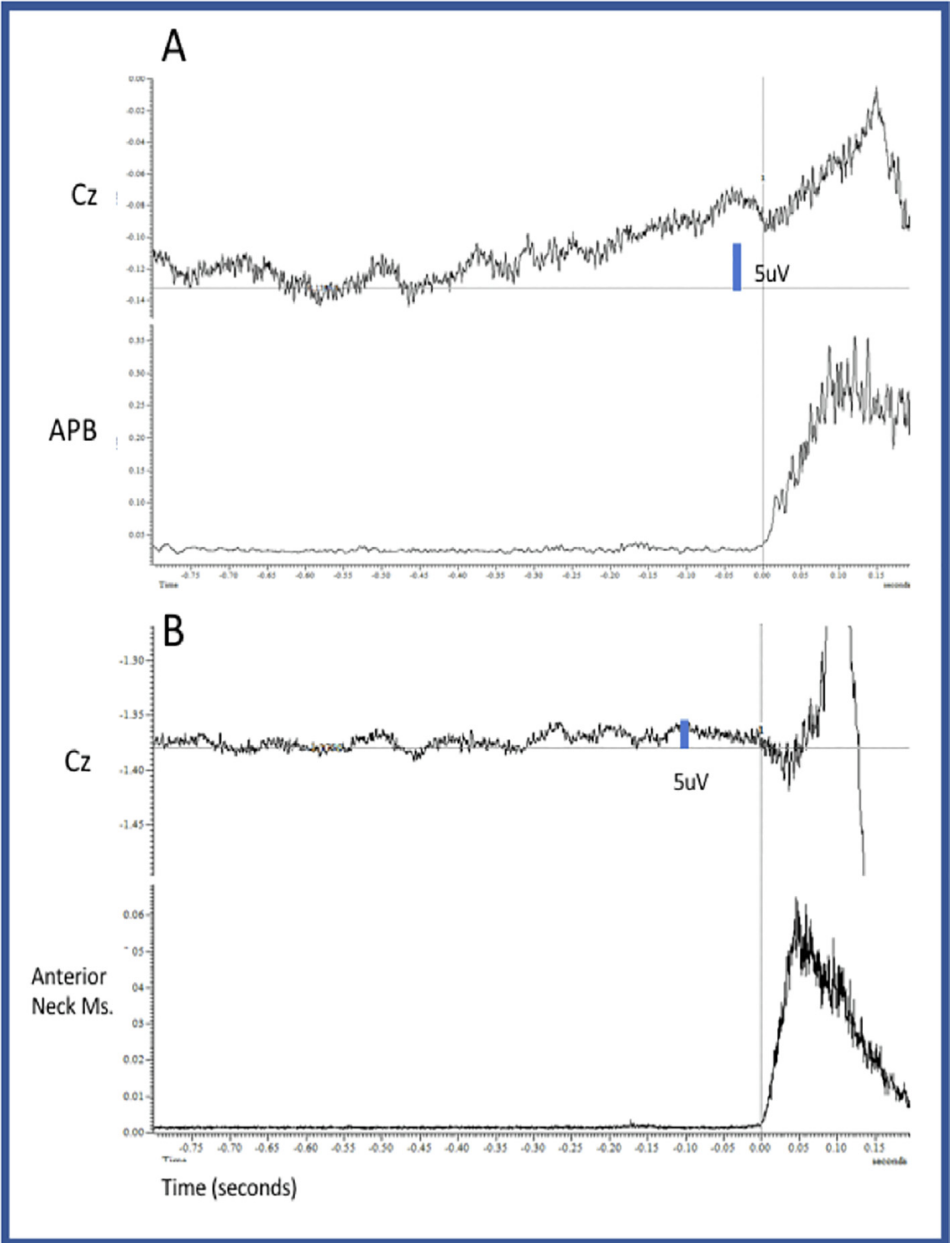
Case 1 BP. A: Back average of voluntary movements. At Cz it is possible to see a clear BP potential that is time locked to the contraction of the APB. B: There is no BP when the back average is done triggered on the anterior neck muscles burst.

BP was found when the patient was asked to do voluntary movement, but it was not present with the involuntary movements of the head and neck (Fig. 2).

The consistent pattern of the movements, lack of entrainment, probable presence during sleep, and the absence of BP were against the diagnosis of FMD. On a follow up visit, patient reported a remote history of a weak sensation of urge which was only present initially during the course of the disease. A diagnosis of tic disorder was made based on the overall synthesis of clinical and electrophysiological data. The patient was not able to tolerate risperidone, but a combination of fluphenazine and botulinum toxin injections helped her symptoms.

### 2.2 Case 2

A 59- year-old right-handed man was referred to our clinic for sudden and brief movements of his trunk. The movements started almost 10 months prior to his presentation. The movements most frequently started from the right side of his back, either lower back or middle back area and moved to the other areas in his back (either right side, left side or both). They would propagate to the lower and upper body parts. He could trigger the movements by pushing over his back at the level of his iliac crest where he reported to have some discomfort. Also sitting with his legs unsupported triggered a more severe set of jerks characterized by flexion of the upper trunk and neck and exhaling and grunting repeatedly. The movements were variable in terms of frequency, intensity and the muscle groups involved. When he was asked to stop the movements, he was able to partially control the movements, but he reported that, “if I try to hold them, it becomes painful.” In addition to the pain and discomfort in the neck and head area, the patient was bothered by the fact that the movements were noticeable by other people. In the beginning, his movements were less frequent and less intense, but they gradually got worse in terms of frequency, amplitude and the initiation sites got more diverse and numerous.

During the evaluation at our clinic, the frequency of his jerky movements differed greatly, between multiple jerks per minute to rarely having one over 10 min.

The history supported a diagnosis of functional movement disorder due to unexplained episodes of improvement and worsening of symptoms and the symptoms not following any known neurologic pattern. On examination, movements demonstrated evidence of distractibility, inconsistency (various muscle groups with various patterns of contraction) and suggestibility (by pushing over a random area on the back symptoms were induced). MRI cervical and lumbar spine demonstrated degenerative spine disease which could not explain the movements initiating from multiple thoracic and non-thoracic muscle groups. An EEG study was normal. Movements were captured during the study and no EEG correlation was evident.

**Fig. 3:**
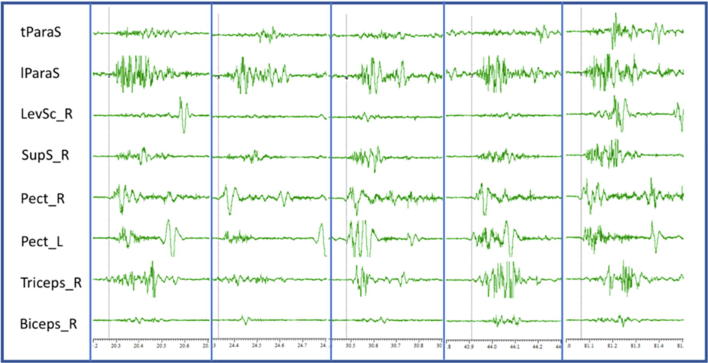
Examples of contractions observed during the recording of case 2. The gray line shows the beginning of the contraction. Although there is a pattern, there is also some variability in the latency of the different muscles. tParaS: thoracic paraspinal, lParaS: Lumbar paraspinal, LevSc_R: Levator scapulae right, SupS_R: Supraspinalis right, Pect_R: Right pectoralis, Pect_L: left pectoralis, Triceps_R: right triceps, Biceps_R: Right biceps.

Surface EMG was recorded from right and left pectoralis major, supraspinalis, elevator scapulae, thoracic and lumbar paraspinals, triceps and biceps. The movement started with right pectoralis contraction with almost simultaneous contraction of left pectoralis (there was some variability, with some movements having up to 15 ms of delay). Between 20 and 30 ms later there was a contraction of the supraspinalis, triceps and the biceps. The muscles of the back had some more variability, but it was important to note that in all the trials, there was initially a contraction of the lumbar paraspinals (in some cases at the same time as the right pectoralis, in other cases up to 30 ms later), followed by thoracic paraspinals going against a rostro-caudal propagation (Fig. 3).

**Fig. 4:**
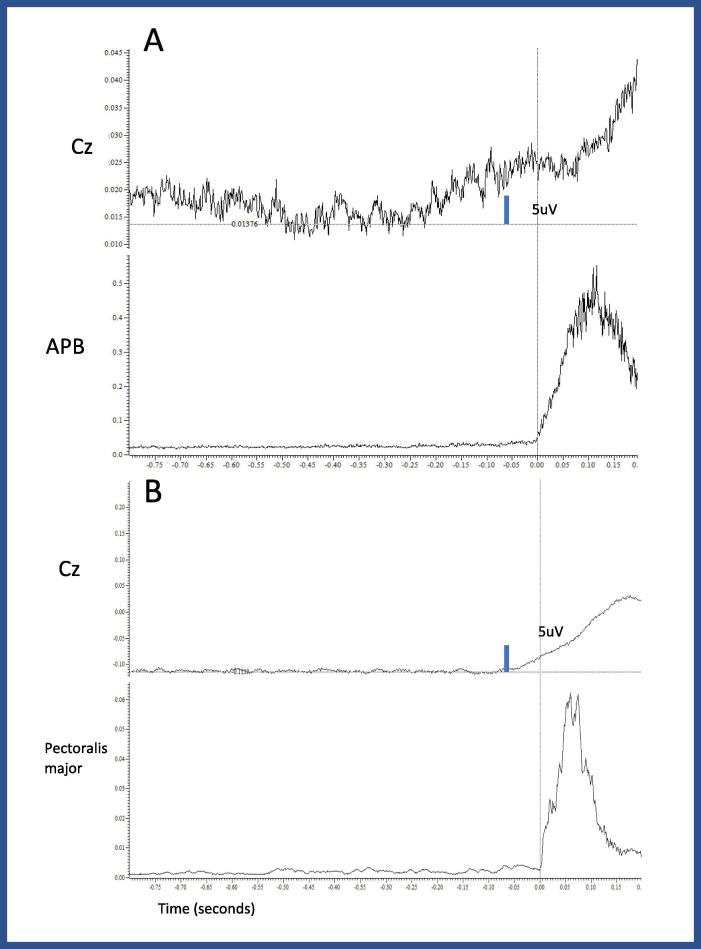
A: Back average of voluntary movements. At Cz it is possible to see a clear BP potential that is time locked to the contraction of the APB. B: There is no BP when the back average is done triggered on the pectoralis major muscle.

In regard to the Bereitschaftspotential, we observed a BP when the patient was asked to perform voluntary movements, but BP was absent prior to the involuntary movements (Fig. 4).

Following the physiologic study, he elaborated further on the discomfort in his back and reported that he has a feeling of tension being built up in his middle back immediately before most of his movements. He reported slight relief of the tension in his back following the jerky movement. A diagnosis of tic disorder was made based on the combined clinical and neurophysiological data. The patient showed some improvement with decreased frequency of his tics after starting guanfacine.

## 3 Discussion

In these two cases with the information acquired on the history and the phenomenology observed on the clinical examination, it was difficult to differentiate FMD versus a tic. The presence of a premonitory sensation is helpful in making the distinction, but sometimes the sensation of urge is not recognized or described by the patient, as was the situation with our patients.

When clinical criteria do not make a clear distinction, polymyographic characterization of the complex movements is a helpful tool in the assessment of movement disorders. It helps to understand the order in which each muscle is activated, the timing of the bursts, and how this pattern changes under different conditions such as sleep and helps also for examining the presence of entrainment and distractibility. Furthermore, it helps to support or reject the findings on the neurological exam.

The Bereitschaftspotential (BP) is a slow negative potential recorded over the bilateral supplementary motor area and premotor cortices finishing with additional activity over the contralateral premotor and motor cortex. It starts 1–1.5 s prior to an intentional voluntary movement. BP has not been demonstrated to precede other movements, including externally triggered voluntary movements (Papa et al., 1991), but functional myoclonus generally has a BP (Hallett, 2010). Physiological studies examining EEG preceding tics show the BP to be uncommon. In one study of six patients with Tourette syndrome, all six patients had BP before voluntarily mimicked tics, but 5 patients had no BP before their tics, and only one patient had a very small BP (late BP) before tics (Obeso et al., 1981). In another set of patients all five patients had BP before voluntarily mimicked tics. Two patients had a late BP before their tics, and three did not have BP before the tics. No correlation between presence of BP and feeling of voluntariness was found in these group of patients (Karp et al., 1996). In a study of 48 patients with jerky movements diagnosed with any of the three conditions psychogenic myoclonus, tic, or organic myoclonus, BP was found to precede the motor tics in a minority of cases. The BP in these patients was shorter in duration in comparison with patients with psychogenic jerks. The authors concluded that the BP is not an ideal ‘gold standard’ test for differentiating psychogenic jerks and motor tics, but it does provide support to the clinical differentiation of the two (van der Salm et al., 2012).

In our first case, the consistency of the pattern of muscle activation was already an argument against a functional disorder. In the second case we did not observe a consistent pattern. Also the age of onset was later than what is usually described for tic disorders. However, both inconsistent pattern and late onset cases of tic disorder have been described in the literature (Baizabal-Carvallo and Jankovic, 2014); (Chouinard and Ford, 2000). In both patients we were able to record BPs when they were doing a voluntary movement. The BP was absent during their involuntary movements. This is an argument against a functional etiology in which the involuntary movements use a similar network as the one used for voluntary movements. As noted, patients with tics usually do not have BP or have only late BP.

The diagnosis of simple tics, particularly with a prominent urge, can be easy, but differentiating complex ones from functional movements is often challenging. Therefore, electrophysiological characterization of the phenomenology and examining the presence of the event related potentials may be helpful in making, or confirming, the final diagnosis. In these two cases, the electrophysiology made us re-think our initial diagnosis therefore proving to be a useful tool. It is clear, however, that further studies are needed for understanding the value, sensitivity, and specificity of these techniques.
